# Supplementary material for: Circulating Tumor Cells and Bevacizumab Pharmacokinetics during Neoadjuvant Treatment Combining Chemotherapy and Bevacizumab for Early Breast Cancer: Ancillary Analysis of the AVASTEM Trial
Source: Cancers (Basel). 2021 Jan 5;13(1):140. doi: 10.3390/cancers13010140 (PMC7796232; doi:10.3390/cancers13010140)
Supplement: Supplementary file 1 [file cancers-13-00140-s001.pdf]

Table S1. Demographics features of patients included in the AVASTEM trial. Data are expressed as N (%) unless otherwise specified.

Clinical Features	All Patients		*p*-Value	CTC Cohort	PK Cohort
	**Bevacizumab (*N* = 50)**	**Control (*N* = 25)**		**All (*N* = 60)**	**Bevacizumab (*N* = 29)**
Age, years (median, min–max)	50.0 (24.3–66.9)	49.8 (28.4–68.5)	0.47	50.6 (24.3–68.5)	50.0 (24.3–66.9)
Menopausal	9 (18)	6 (24)	0.55	13 (22)	9 (31)
Tumor size T2	21 (42)	10 (40)	1	23 (38)	15 (52)
Tumor size T3	17 (34)	9 (36)		19 (32)	9 (31)
Tumor size T4	12 (24)	6 (24)		18 (30)	5 (17)
Positive axillary lymph node	37 (75.5)	22 (88)	0.24	48 (81)	21 (75)
De novo metastatic	7 (14)	3 (12)	1	8 (13)	5 (17)
